# Evolutionary history and phylogeographic relationships of shrews from *Sorex araneus* group

**DOI:** 10.1371/journal.pone.0179760

**Published:** 2017-06-26

**Authors:** Paweł Mackiewicz, Magdalena Moska, Heliodor Wierzbicki, Przemysław Gagat, Dorota Mackiewicz

**Affiliations:** 1 Department of Genomics, Faculty of Biotechnology, University of Wrocław, Wrocław, Poland; 2 Department of Genetics, Wroclaw University of Environmental and Life Sciences, Wroclaw, Poland; University of Innsbruck, AUSTRIA

## Abstract

Shrews of the *Sorex* genus are an evolutionarily successful group that includes more than 77 species widely distributed in Eurasia and North America. The genus is one of the rare cases where karyotypic changes reflect well the evolutionary relationships among its species. The taxa showing the greatest variation in karyotype are usually classified into the *Sorex araneus* group. Its evolution was associated with chromosomal rearrangements, which could have promoted fast diversification of this group into many chromosomal races and species. These processes were additionally complicated by introgressions of mitochondrial DNA, which made the evolutionary history of this group quite complex and difficult to infer. To tackle the problem, we performed multi-method phylogenetic analyses based on mitochondrial cytochrome b that is considered a good molecular marker available for many representatives of *Sorex*. The results were compared with phylogenies based on chromosomal rearrangement data and put into temporal and spatial context using molecular dating and historical biogeography methods. We complemented the study with the estimation of diversification rates within the *S*. *araneus* group as well as comparing the results with paleontological records and climatic oscillations within the last 4 million years. Based on the gathered data, we proposed a hypothetical scenario for the evolution and geographic dispersion of species belonging to the *S*. *araneus* group. The shrews began to diversify about 2.7 million years ago in Eurasia and then migrated at least twice to North America. The evolution of shrews was driven by Pleistocene glacial and interglacial cycles, which increased their speciation rate and the emergence of new lineages. The migrations of populations were accompanied by introgressions of mitochondrial DNA into native shrews and occurred at least twice.

## Introduction

**Fig 1:**
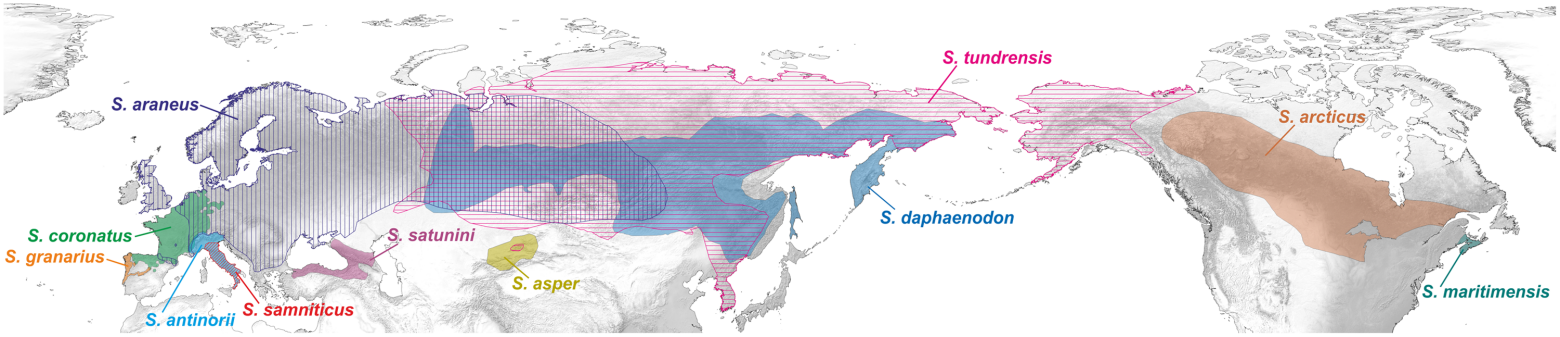
Current distribution of selected species belonging to *Sorex araneus* group. The data were obtained from the IUCN Red List of Threatened Species. Version 2014.1. http://www.iucnredlist.org. Downloaded on 28 March 2015.

Shrews of the *Sorex* genus, though very common, are unusual because their complex evolution can be deduced from analyses of their karyotypes. This genus has had great evolutionary success and currently includes more than 77 species widely distributed in Eurasia and North America [1]. Cytogenetic studies recognized within this genus a large group of species where males are characterized by XY_1_Y_2_ sex chromosome system with the compound X chromosome derived from the centric fusion of an autosome and an X [2–6]. This group is called either *araneus-arcticus* group [5–14] or *araneus* group [15–21] after its typical representatives. It comprises 12 species [1, 21] that inhabit the Holarctic, from Western Europe across Asia to the east coast of North America. Molecular studies of allozymes [22] and mitochondrial cytochrome b confirmed monophyly of this clade [11, 23]. A sister lineage to the *S*. *araneus* group is the Apennine shrew *S*. *samniticus* [18], which is endemic to the Italian Peninsula (Fig 1). Although this shrew lacks the XY_1_Y_2_ system, it shows considerable homologies in karyotype with representatives of the *S*. *araneus* group [2, 24–26].

In comparison to other shrews, the *S*. *araneus* group revealed a higher than expected rate of cladogenesis [23]. It can be related to the extraordinary variation in karyotypes that may promote rapid differentiation of populations into many chromosomal races and species [21, 27–29]. These species and races were recognized based on unique chromosomal segments and karyotypic features. The extensive inter- and intraspecific chromosomal variation results from Robertsonian rearrangements accompanied by telomere–centromere tandem translocations, centromere shifts and pericentric inversions [5, 19].

The best known representative of the *araneus* group is the common or Eurasian shrew, *S*. *araneus sensu stricto* that is widely distributed in the Palaearctic, from Britain through Europe to Asia, as far as Lake Baikal and the Arctic coast (Fig 1). Its close relatives include: (1) the Siberian large-toothed shrew, *S*. *daphaenodon*, distributed from Ural Mountains to the coast of the Pacific Ocean, in the Russian Federation, Mongolia, north-eastern China and North Korea; (2) the Caucasian shrew, *S*. *satunini*, found in northern Turkey and the Caucasus; (3) the crowned (Millet's) shrew, *S*. *coronatus*, occurring from northern Spain and France to the Low Countries, northern Switzerland, Germany and Austria, as well as on the Jersey island (United Kingdom); (4) the Valais shrew, *S*. *antinorii* inhabiting Italy, south-eastern France and southern Switzerland; and (5) the Iberian shrew, *S*. *granarius*, found in Portugal and Spain. The latter is considered a sister lineage to the other members of *S*. *araneus* group because the ancestral karyotype of this group is assumed to consist of un-rearranged acrocentric chromosomes, which are just found in *S*. *granarius* [5, 14, 30].

The other evolutionary lineages of the *S*. *araneus* group are: (1) the Arctic shrew, *S*. *arcticus*, present in Canada and the northern United States; (2) the maritime shrew, *S*. *maritimensis*, restricted to Nova Scotia and New Brunswick in Canada; (3) the tundra shrew, *S*. *tundrensis*, widely ranging from northwest Canada and Alaska through Russia to the Urals and Altai Mountains; and (4) the Tien Shan shrew, *S*. *asper* occurring in a limited range of northwest China, southeast Kazakhstan and northeast Kyrgyzstan (Fig 1).

The *S*. *araneus* group also includes: (1) the Udine shrew (*S*. *arunchi*), restricted to the Udine province of north-east Italy and western Slovenia, and (2) the Gansu shrew (*S*. *cansulus*) known only from the Gansu province in China. The former seems to be related or conspecific to *S*. *antinorii* [31, 32] and the latter to *S*. *tundrensis* [1, 33]. Since neither their karyotypes nor cytochrome b sequences were obtained, these species were not included in the study.

Different analyses based on biochemical, karyotypic, and sequence data were carried out to propose evolutionary relationships between taxa belonging to the *S*. *araneus* group. Some of them were done in the context of global *Sorex* phylogeny [11, 23], whereas others focused on selected members and subgroups [9, 12–14, 16, 18, 19, 31, 34–39]. However, the results based on the various types of data were often contradictory [12, 16, 34, 37] and they were explained by hybridization and/or introgression between some taxa, e.g. *S*. *antinorii* and the Cordon chromosomal race of *S*. *araneus* [40–44], *S*. *granarius* and the Carlit race of *S*. *araneus* [34, 37] as well as *S*. *satunini* and an ancestor of *S*. *araneus* [12, 36]. These processes make the evolutionary history of the *Sorex araneus* group quite complex but very interesting to investigate.

To tackle the problem of the evolutionary history of *S*. *araneus* group, we carried out comprehensive phylogenetic analyses based on mitochondrial DNA and confronted them with karyotype data to reconcile their results. The phylogeny was placed within a temporal and spatial framework using molecular dating and methods reconstructing ancestral geographic distribution. Moreover, we calculated diversification rates of shrew lineages in time and correlated them with climatic changes and fossil records. Taking together all the available data, we propose a hypothetical scenario for the evolution and geographic dispersion of taxa belonging to the *Sorex araneus* group.

## Material and methods

### Sequence data sets

Mitochondrial cytochrome b sequences of *Sorex araneus* group and outgroup species were thoroughly collected from GenBank using BLAST searches. Analyses were performed on three alignment sets including: (1) 111 representative sequences of *Sorex araneus* group and outgroup species (with the length of 1011 bp); (2) 140 sequences from *S*. *araneus* and four from *S*. *coronatus* used as an outgroup (1011 bp); and (3) 93 sequences from *S*. *araneus* and one from *S*. *coronatus* used as an outgroup (1140 bp). Since some species, such as *S*. *antinorii*, *S*. *araneus* and *S*. *tundrensis* were overrepresented by more than 100 sequences in comparison to other taxa, we reduced their sets to 20 most informative sequences using T-Coffee [45].

### Phylogenetic analyses

Phylogenetic trees were inferred in four programs and seven approaches: Bayesian inference (BI) with MrBayes [46], maximum likelihood (ML) with TreeFinder [47, 48] and morePhyML [49, 50] as well as neighbour joining (NJ), minimum evolution (ME), weighted least squares (WLS) and maximum likelihood (ML) with PAUP* 4.0b [51]. All these methods were applied in the case of the first alignment (see section Sequence data sets), whereas ME, WLS and ML methods with PAUP were omitted in the second and third alignments focused on *S*. *araneus*.

In MrBayes analyses, we applied three separate nucleotide substitution models mixed+Γ+I for three codon positions. We applied two independent runs starting from random trees using 8 (for the first alignment) and 32 (the two other alignments) Markov chains. Trees were sampled every 100 generations for 10,000,000 generations. Finally, we selected trees from the last 2,509,000 to 3,397,000 generations (depending on the alignment) that reached the stationary phase and convergence. In TreeFinder, we also applied separate substitution models for three codon positions, according to the Propose Model module (S1 Table). The ML trees constructed with morePhyML and PAUP as well as trees based on three distance methods (NJ, ME and WLS) were calculated using the best-fit substitution model proposed in jModeltest among 1624 candidates [52] (S1 Table).

We used search depth = 2 in TreeFinder as well as NNI and SPR algorithms in morePhyML. In ML, ME and WLS methods, final trees were searched from 10 starting trees obtained by stepwise addition with random-addition of sequences followed by TBR algorithm. Bootstrap analyses were performed on 1000 replicates. Additionally, we applied the Local Rearrangements-Expected Likelihood Weights (LR-EWL) method in TreeFinder and the approximate likelihood ratio test (aLRT) based on a Shimodaira-Hasegawa-like procedure in morePhyML [53]. Among-site rate variation was modelled with the gamma distribution with five category rates.

Tests of tree topologies were carried out in Consel [54] assuming 1,000,000 replicates based on site-wise log-likelihoods calculated in TreeFinder under the best fitted substitution models found in this program. Competitive topologies obtained in MrBayes were compared using Bayes Factor based on the stepping-stone method estimating the mean marginal likelihood from 4 independent runs using 8 Markov chains, 50 steps of the sampling algorithm and 10,000,000 generations of the MCMC simulation.

### Species delimitation studies

To delimit shrew species, we used the Automatic Barcoding Gap Detection (ABGD) method [55] and the General Mixed Yule-Coalescent model (GMYC) [56, 57]. ABGD was carried using a distance matrix obtained from the MrBayes phylogenetic tree. GMYC analyses were performed in the R environment [58] using the Splits package [59]. We applied both single [56] and multiple threshold models [57]. The input tree was obtained by the conversion of the MrBayes tree to the ultrametric one using chronopl command from Ape package in R [60], which implements the penalized likelihood method [61].

### Molecular dating

Divergence times were estimated using Beast software [62]. The best-fit substitution models were selected among 1624 candidate models in jModeltest, separately for three codon positions (S1 Table). We applied an exponential prior on the divergence age of *S*. *daphaenodon* assuming the minimum bound of 0.8 million years ago (Mya) according to its earliest fossils dated to 0.8–0.95 Mya [63, 64]. This assumption agrees with the molecular clock analyses that suggested the divergence time between *S*. *araneus* and *S*. *daphaenodon* clades about 1 Mya [39]. The other calibration point assumed the minimum bound of 0.36 Mya in an exponential prior on the divergence age of *S*. *satunini*, whose earliest fossils were found in the Northern Caucasus in Middle and Late Pleistocene layers, dated to 0.36 Mya [65, 66].

We applied the calibrated Yule model and tested strict as well as lognormal relaxed clock models for the three codon positions separately. The mutation rate for the third codon position was estimated based on uniform distribution using initial value 0.055 with upper and lower limits of 0.028 and 0.089 per million years according to the results obtained for *Sorex* by Hope et al. [67] and [13]. These values correspond well to the other estimation by Yannic et al. [68], i.e. 0.071 per million years with a 95% confidence interval of 0.057–0.085. The initial values of the mutation rate for the first and second codon positions were appropriately rescaled according to substitution rates obtained for the three codon positions in TreeFinder. Finally, we applied the separate relaxed clock models for all codon positions because the standard deviation of the uncorrelated lognormal relaxed clock exceeded one indicating a significant variation among branches. The posterior distributions of parameters were estimated for 100,000,000 generations with the sampling frequency of 1000 steps. After checking convergence and sufficient sampling in Tracer [69], phylogenetic trees were summarized in TreeAnnotator [62] to the maximum clade credibility tree with common ancestor heights using 10% burn-in of all trees. The generated tree was visualized in FigTree [70].

### Diversification rate estimation

The maximum clade credibility tree calculated in Beast with associated branching times was used to estimate the diversification rate in R package with LASER 2.4 [71]. To test whether diversification rates decreased with time, we calculated the γ statistic [72]. We tested 11 likelihood models for diversification rates [73, 74]—see S2 Table for details. The models were compared according to the Akaike information criterion (AIC). To visualize the variation of diversification rates with time, the rates were calculated within overlapping periods of 400 thousand years using yuleWindow function [75] and compared with the δ^18^O curve [76], which is a good climate proxy. For the comparison and better visualization of climate oscillations, we calculated the variance in the δ^18^O records within the same overlapping periods.

### Phylogeny based on chromosomal rearrangements

Ten representatives of *Sorex*: *S*. *araneus* Cordon race, *S*. *antinorii*, *S*. *coronatus*, *S*. *daphaenodon*, *S*. *granarius*, *S*. *maritimensis*, *S*. *samniticus*, *S*. *satunini*, *S*. *tundrensis* from Kemerovo and Tomsk were compared in terms of chromosomal whole-arm rearrangements. The Cordon race was selected as the representative of *S*. *araneus* because it shows the most primitive karyotype, which is characterized by polymorphic metacentric fusion j:l and more acrocentric chromosomes than other races [30, 35]. The data concerning the rearrangements and chromosomal characters was taken from appropriate references [9, 16, 77–79], including the newest one based on Zoo-FISH hybridization [19]. We analysed 29 available chromosomal rearrangement characters by maximum parsimony approach in PAUP and Bayesian in MrBayes. We coded them into three states: the presence, absence and intermediate state that represents unfixed or polymorphic state. The characters were considered as ordered and unordered (in MrBayes and PAUP) as well as irreversible assuming an ancestor with unarranged states (in PAUP). All possible 2,027,025 trees for 10 taxa were evaluated in PAUP. We assumed 1,000,000 replicates in bootstrap analyses and searched trees from starting trees obtained by the simple stepwise addition of characters’ sequences followed by TBR algorithm. In MrBayes, we applied two independent runs starting from random trees using 16 Markov chains. Trees were sampled every 100 generations for 50,000,000 generations. Finally, we selected trees from the last 24,304,000 generations that reached the stationary phase and convergence.

### Historical biogeography

In order to reconstruct the ancestral geographic distributions of *S*. *araneus* taxa, we carried out likelihood analysis of geographical range evolution (Lagrange) using the dispersal-extinction cladogenesis (DEC) model [80] implemented in the RASP 3.2 (Reconstruct Ancestral State in Phylogenies) tool [81]. The study was based on the condensed tree inferred in Beast. We considered the tree in which *S*. *granarius* was placed as the basal lineage to the other members of *S*. *araneus*, i.e. before the divergence of *S*. *daphaenodon*, as was indicated by the phylogeny based on chromosomal rearrangements. In the model, we determined ten biogeographic regions which are inhabited currently by shrews of the *S*. *araneus* group: Eastern, Central and Western America, Northern and Central Asia, Eastern and Western Europe, Caucasus as well as Apennine and Iberian Peninsula. We assumed up to three areas in the ancestral distributions. Range and dispersal constraints were set according to the adjacency of these regions.

## Results

### Phylogenetic relationships based on mtDNA

**Fig 2:**
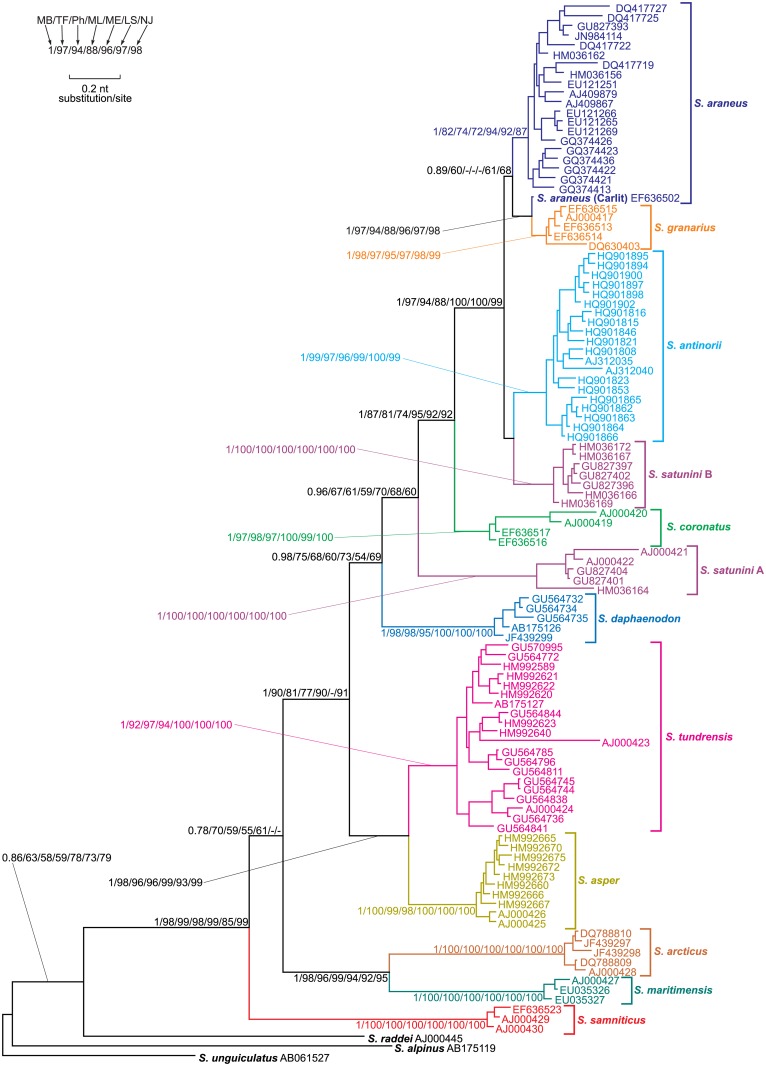
MrBayes tree for the alignment of cytochrome b sequences from *Sorex araneus* group and the outgroup. Numbers at nodes, in the order shown, correspond to: posterior probabilities estimated in MrBayes (MB) as well as bootstrap values obtained in TreeFinder (TF), PhyML (Ph) and PAUP by maximum likelihood (ML), minimum evolution (ME), weighted least squares (LS) and neighbour joining (NJ) methods. Values of the posterior probabilities and bootstrap percentages lower than 0.50 and 50%, respectively, were omitted or indicated by a dash "-".

To determine phylogenetic relationships within the *S*. *araneus* group, we applied seven approaches based on a popular mitochondrial marker cytochrome b. All methods produced very similar and resolved trees with moderately to very well-supported clades (Fig 2). The earliest diverging lineage sister to this group is *S*. *samniticus*. Both lineages create a monophyletic clade with very high support values, distinctly separated from the outgroup. Although the *S*. *araneus* group did not obtain such strong support, it was reproduced by all methods used. The two subsequently branching off lineages include: (1) *S*. *maritimensis* and *S*. *arcticus*, as well as (2) *S*. *asper* and *S*. *tundrensis*. Both of them are significantly supported by all methods.

The other taxa create a monophyletic clade including *S*. *araneus* and closely related species, which is very significantly supported by the Bayesian approach and moderately by other methods (Fig 2). *S*. *daphaenodon* is the earliest branch of this group and its sister lineage was inferred by all methods with rather moderate support. The next diverged lineage includes sequences representing the Transcaucasian population of *S*. *satunini* with the cytochrome b haplotype A [36]. Its sister clade was reproduced by all methods and obtained very high support by Bayesian approach and three distance methods. This clade consists of *S*. *coronatus* and a very significantly supported lineage including four other *Sorex* species. Two of them, *S*. *antinorii* and *S*. *satunini* from Northern Ciscaucasia described as *tembotovi* subspecies [82] with the cytochrome b haplotype B [36], are clustered together. However, this grouping did not obtain posterior probability, p-values or bootstrap values larger than 0.5 or 50% in any methods, although it was inferred by 5 of 7 methods. Two unpartitioned ML methods in morePhyML and PAUP clustered *S*. *satunini* haplotype B with the clade including all *S*. *araneus* and *S*. *granarius* samples. These two species create a much better supported clade. Although some methods did not produce bootstrap values larger than 50%, this group was present in all trees obtained by seven methods. However, not all samples of *S*. *araneus* are monophyletic because the sequence representing the Carlit race is grouped very significantly with *S*. *granarius* but not with other *S*. *araneus* sequences, which may result from the introgression of mtDNA.

S1 and S2 Figs present detailed phylogenetic relationships between all available sequences from *S*. *araneus*, with lengths of 1011 bp and 1140 bp, respectively. The obtained trees for both sets, especially internal branches, were not well-resolved. However, some clades were inferred by all four methods used and some clusters can be recognized. Many samples from Russia are placed at the basal position of the tree based on the most taxon-rich alignment (S1 Fig). Most sequences from Poland are grouped to one clade but without any significant support. Clades containing samples exclusively from one region, i.e. Hungary, Scotland or Sweden are also present. However, other sequences do not show any geographic pattern. Several clades that were found by all applied methods include sequences from distant regions, i.e. Scotland and Hungary as well as Scotland and Switzerland. In the tree for the full length alignment of cytochrome b (S2 Fig), the basal position is occupied by samples from Finland and also Russia. This tree includes one relatively strongly supported clade of Scotland samples.

When it was possible, we assigned names of races and karyotypic groups to samples. However, we did not observe any significant clustering of the races or the groups of the same type. It is visible especially well in the case of samples from Poland, for which East European and West European karyotypic groups are intermixed (S1 Fig).

### Testing the phylogenetic position of *S*. *satunini*

**Fig 3:**
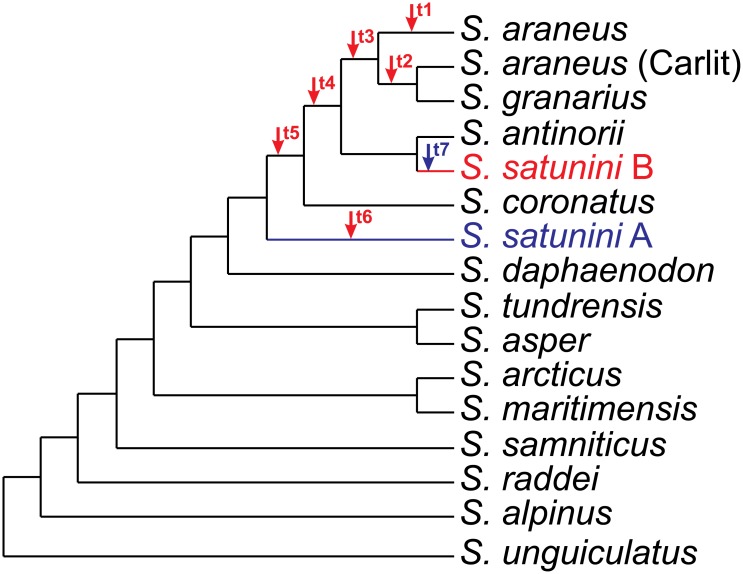
Alternative positions of *S*. *satunini* haplotypes in the best tree found in MrBayes and TreeFinder. The alternative position of *S*. *satunini* haplotype B is indicated by red arrows (t1 –t6) and the position of *S*. *satunini* haplotype A is marked by a blue arrow (t7). Results of tests comparing these topologies are shown in Table 1.

The position of *S*. *satunini* with the haplotype B of cytochrome b is most controversial in the tree of *S*. *araneus* group and may result from the introgression of mtDNA (Fig 2). Therefore, we tested its alternative placements in the tree to check competitive phylogenetic hypotheses (Fig 3, Table 1).

**Table 1:** Results of tests comparing tree topologies presented in Fig 3.

Tree	AU	NP	BP	PP	SH	wSH	BF
t0	0.605	0.375	0.373	0.428	0.874	0.875	0.00
t1	0.066	**0.032**	**0.031**	**0.006**	0.536	0.564	**4.52**
t2	0.258	0.15	0.106	**0.01**	0.568	0.598	**3.26**
t3	0.582	0.118	0.119	0.144	0.891	0.866	0.81
t4	0.644	0.360	0.360	0.412	0.930	0.929	0.14
t5	**0.020**	**0.010**	**0.010**	**8.0E-07**	0.120	0.077	**16.31**
t6	**0.003**	**0.001**	**0.001**	**2.0E-11**	**0.028**	**0.023**	**27.88**
t7	**0.002**	**3.0E-04**	**3.0E-04**	**6.0E-10**	**0.019**	**0.023**	**23.67**

The topology t0 corresponds to the MrBayes tree shown in Fig 2. The table includes: p-values from an approximately unbiased test (AU), bootstrap probabilities calculated from all sets of scaled replicates (NP) and from one set of replicates (BP), Bayesian posterior probabilities calculated by BIC approximation (PP), p-values from Shimodaira-Hasegawa (SH) and weighted Shimodaira-Hasegawa (wSH) tests, and a Bayes factor (BF) expressed as differences in natural logarithm likelihood units from the best topology (t0). Probabilities and p-values < 0.05, and BF > 3 were **bolded**.

The placement of haplotype B as a sister clade to the group of *S*. *granarius* with *S*. *araneus* Carlit race (topology t2) and the clade comprising other *S*. *araneus* samples (topology t1) were rejected by two and four test, respectively. The location of this haplotype before the divergence of *S*. *coronatus*, *S*. *antinorii*, *S*. *granarius* and *S*. *araneus* (topology t5) is less likely and rejected by five tests. Only two positions were not significantly worse than the best tree topology: the grouping of the haplotype B with the whole clade of *S*. *granarius* and *S*. *araneus* (topology t3), and its clustering with the latter clade, including in addition, *S*. *antinorii* (topology t4). Both hypotheses assuming the monophyly of the two *S*. *satunini* haplotypes (topologies t6 and t7) were significantly rejected by all tests applied.

### Delimitation of species boundaries

The taxonomic status of members within the *S*. *araneus* group was revised several times. *S*. *tundrensis* was initially considered a subspecies of *S*. *arcticus* and obtained species status after morphometric [64] and genetic studies [8, 23]. Similarly, *S*. *maritimensis* was included within *S*. *arcticus* as a subspecies until it was elevated to species status [1, 38]. *S*. *antinorii* was also formerly known as the Valais chromosome race of *S*. *araneus* and the species rank was obtained after Brunner et al. [31] revision. Therefore, it seems interesting to check how various delimitation methods based on mitochondrial cytochrome b recognize species within one set including many representatives of the *S*. *araneus* group. It may also help to recognize the introgression of mtDNA.

**Fig 4:**
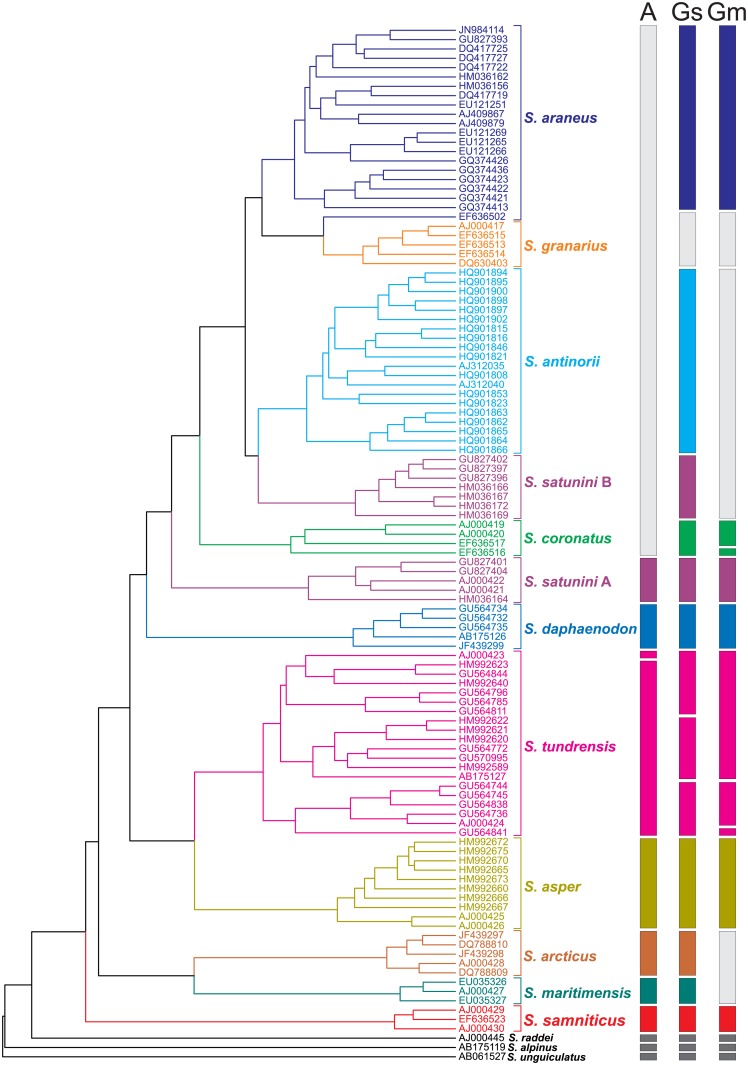
*Sorex* species delimitation. The clusters of delimited species were obtained with ABGD (A) method as well as GMYC single- (Gs) and multiple-threshold (Gm) models.

We applied ABGD and GMYC methods (Fig 4). Both GMYC models assuming single- and multiple-threshold were fitted to the data significantly better (Likelihood Ratio test, p-values 0.0027 and 2.8·10^−5^, respectively) than the null model assuming that the entire sample comes from a single species with uniform branching. The single GMYC method recognized 17 potential species, the multiple-threshold GMYC method 16 species and the ABGD method found 12 species. The results of various ABGD analyses were consistent and did not depend on an *a priori* threshold value on the maximal distance.

Three outgroup shrews were identified as separate species in all three approaches (Fig 4). Well established species, such as: *S*. *samniticus*, *S*. *asper*, *S*. *daphaenodon* and the *S*. *satunini* haplotype A were also recognized as separate clusters. The ABGD and single GMYC methods separated *S*. *arcticus* and *S*. *maritimensis* but the multiple GMYC considered them as one species, which agrees with the former classification, when *S*. *maritimensis* was the subspecies of *S*. *arcticus*. The recognition of species within *S*. *araneus* and closely related taxa is the most variable. The ABGD produced one cluster including *S*. *coronatus*, the haplotype B of *S*. *satunini*, *S*. *antinorii*, *S*. *granarius* and *S*. *araneus*. The GMYC methods discriminated *S*. *granarius* together with *S*. *araneus* Carlit race as one cluster and the other *S*. *araneus* as the second one. The *S*. *satunini* haplotype B and *S*. *antinorii* were also separated by the single GMYC method but the multiple GMYC clustered them into one group. The single GMYC method assigned all samples of *S*. *coronatus* to the separate cluster, whereas the multiple GMYC separated the most diverged basal sequence as a singleton.

Interestingly, *S*. *tundrensis* was not recognized as one group but was split into several clusters (Fig 4). The separation into three clusters according to the single GMYC method is associated with phylogeographic differentiation of this shrew into populations inhabiting (1) West Siberia, (2) East Asia as well as (3) Far West Siberia and Nearctic region [13] or (1) Central Asia, (2) East Asia as well as (3) West Asia with Nearctic region [39].

### Divergence time and diversification rate studies

**Fig 5:**
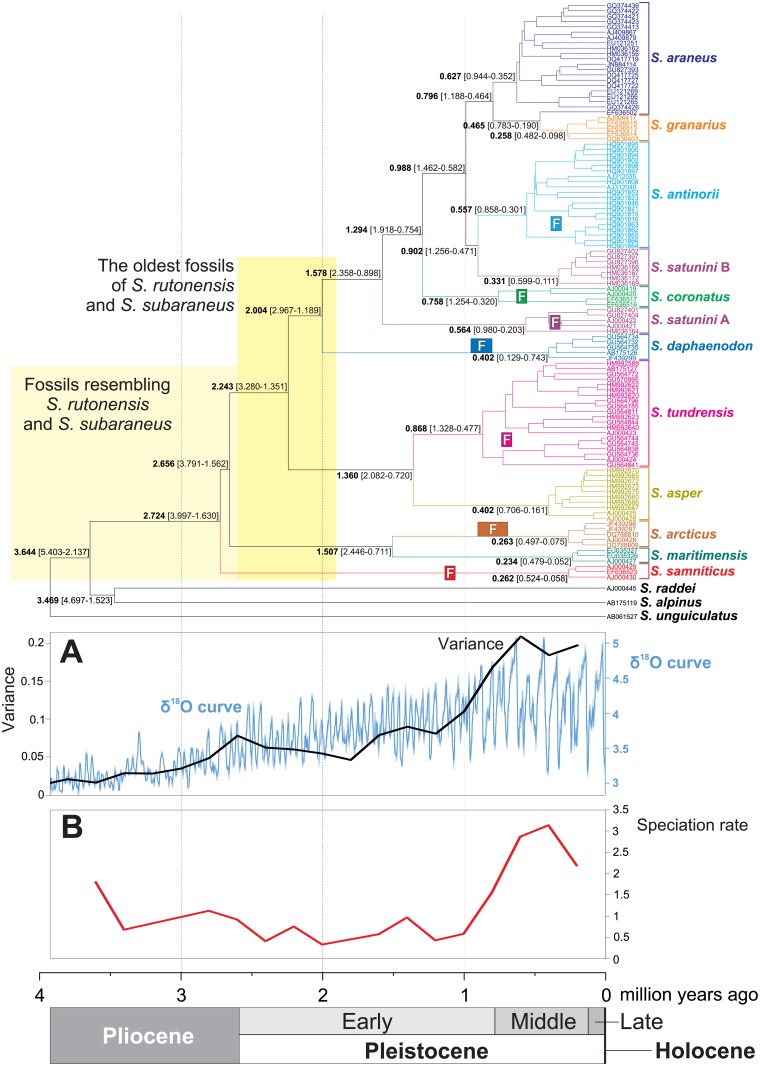
Chronogram for *Sorex araneus* group and its closely related taxa. The chronogram is the maximum clade credibility tree obtained in Beast for the alignment of cytochrome b sequences from *Sorex araneus* group and its closely related taxa. Mean ages (in bold) as well as the 95% highest posterior confidence interval (in parenthesis) are shown for main nodes. The tree was compared with the benthic δ^18^O curve according to Lisiecki and Raymo [76] and its variance (A) as well as the rate of new lineages’ origin was calculated within overlapping periods of 400 thousand years (B). The oldest known fossils of some lineages were indicated (F)–see S3 Table for details.

In order to place the phylogeny of *Sorex araneus* mitochondrial lineages in the temporal framework, we performed molecular dating analyses (Fig 5). They showed that the diversification of these lineages started at the Pliocene/Pleistocene boundary about 2.7 Mya (million years ago), when the *S*. *samniticus* lineage was separated. North American shrews split into *S*. *maritimensis* and *S*. *arcticus* about 1.5 Mya, and Asiatic shrews diverged into *S*. *asper* and *S*. *tundrensis* about 1.4 Mya. The successive diversification of *S*. *araneus* and closely related taxa started about 2 Mya through the separation of the *S*. *daphaenodon* lineage. The radiation of *S*. *araneus* began around 0.800 Mya. The origin of mitochondrial lineages represented by the *S*. *satunini* haplotype B and *S*. *granarius* are dated to ca. 0.900 Mya and 0.470 Mya, respectively.

Using diversification rate analyses we checked if and when shrews were subjected to an increased speciation rate. The calculated γ statistics (2.322, p-value = 0.9899) gives no evidence for the significant slowdown in the diversification. Among tested diversification models, yule4rate turned out the best-fitting with the lowest AIC (S2 Table). The model proposed three shifts in the speciation rate. The rapid increase in the speciation rate (from 0.662 to 2.993) is dated to 0.796 Mya. Up to this time, the rate was rather stable, which is also visible in the variation of diversification rate calculated within overlapping periods of 400 thousand years (Fig 5B). The increase in the diversification rate is associated with the emergence of many lineages of *S*. *tundrensis* and the other members of *S*. *araneus* group. However, around 130 kya (thousand years ago), a decrease in this rate transpired (to 1.433), which further declined ca. 48 kya (to 0.375) (see S2 Table). Interestingly, the time of the largest diversification (Fig 5B) corresponds well to the most intensive climate fluctuations in the Pleistocene, which started about 2 Mya (Fig 5A). In fact, we found a significant positive Pearson correlation coefficient = 0.923 (p-value = 0.0004) between the speciation rate and the variance in the climate fluctuations based on the δ^18^O curve, in the period from 2 Mya up to the present day.

### Phylogenetic relationships based on karyotypic data

**Fig 6:**
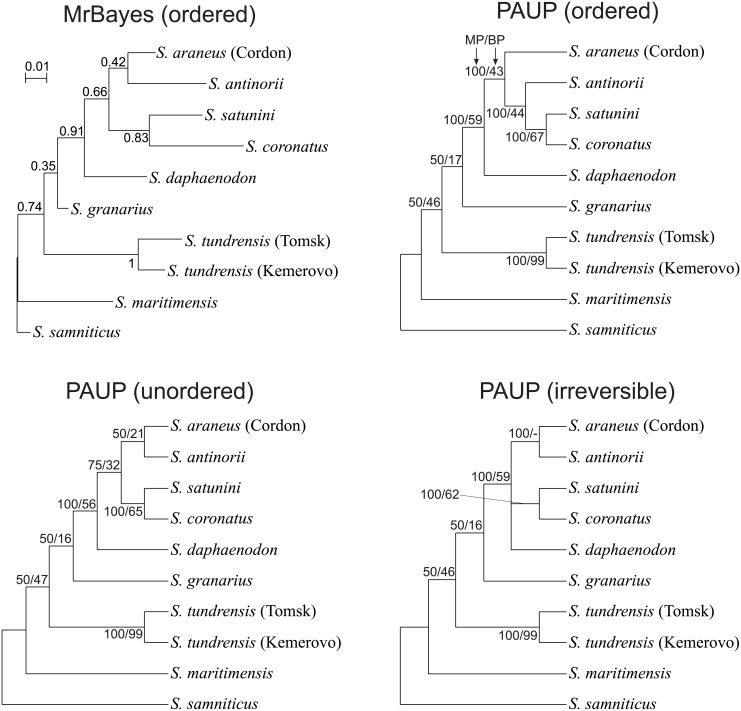
MrBayes and PAUP consensus trees based on karyotypic characters considered as ordered, unordered or irreversible. Numbers at nodes in the MrBayes tree indicate posterior probabilities, whereas in the PAUP trees, the percentage of maximum parsimony trees supporting the given clade (MP) and bootstrap percentages (BP). The MrBayes tree for unordered characters has the same topology and almost the same posterior probabilities as the tree for ordered characters (shown in the figure).

Using chromosomal whole-arm rearrangement data available for ten representatives of *Sorex*, we reconstructed their relationships using Bayesian (BA) and maximum parsimony (MP) (Fig 6) methods. The obtained trees produced very similar branching orders. As an outgroup, we chose *S*. *samniticus* characterized by not-rearranged fully acrocentric chromosomes in comparison to *S*. *araneus* representatives [2, 24–26]. The next diverged lineage is *S*. *maritimensis* and then two representatives of *S*. *tundrensis* from different localities and with different chromosome G-banding [19]. In one half of the consensus MP trees *S*. *maritimensis* and *S*. *tundrensis* were separated, whereas in the other half they were grouped in one clade (Fig 6). *S*. *maritimensis* share with *S*. *tundrensis* f+o fusion but possess four other unique fusions (b+n, n+t, o+u, g+r) and lacks t+u fusion, which is a common feature of *S*. *tundrensis* and all other *S*. *araneus* representatives. The two *S*. *tundrensis* differ by three unique fusions c+l, h+m, and h+p [19].

In contrast to the mitochondrial phylogenetic tree, the basal position to the other members of *S*. *araneus* and closely related taxa is occupied by *S*. *granarius*, which besides the t+u fissions does not have any other chromosome rearrangements (e.g. a+f fusion) typical of subsequently diverged members. The common grouping of these members is supported by BA and all consensus MP trees. The next separated lineage is *S*. *daphaenodon*, which apart from the a+f and t+u fusions, has three other unique ones (b+h, c+g, l+m). All trees with moderate support clustered *S*. *satunini* and *S*. *coronatus* sharing a unique j+n fusion. The position of *S*. *antinorii* is less stable. In BA and two MP trees, *S*. *antinorii* groups with the Cordon race. However, in the MP trees assuming ordered characters *S*. *antinorii* clusters with the clade *S*. *satunini* + *S*. *coronatus*. This inconsistency may result from the exclusive presence of l+o fusion in *S*. *satunini* and *S*. *coronatus* in a monomorphic state and in *S*. *antinorii* in a polymorphic state. Simultaneously, the *S*. *araneus*, *S*. *antinorii* and *S*. *satunini* are characterized by b+c fusion, which is absent from *S*. *coronatus*.

### Reconstruction of ancestral geographic distributions

**Fig 7:**
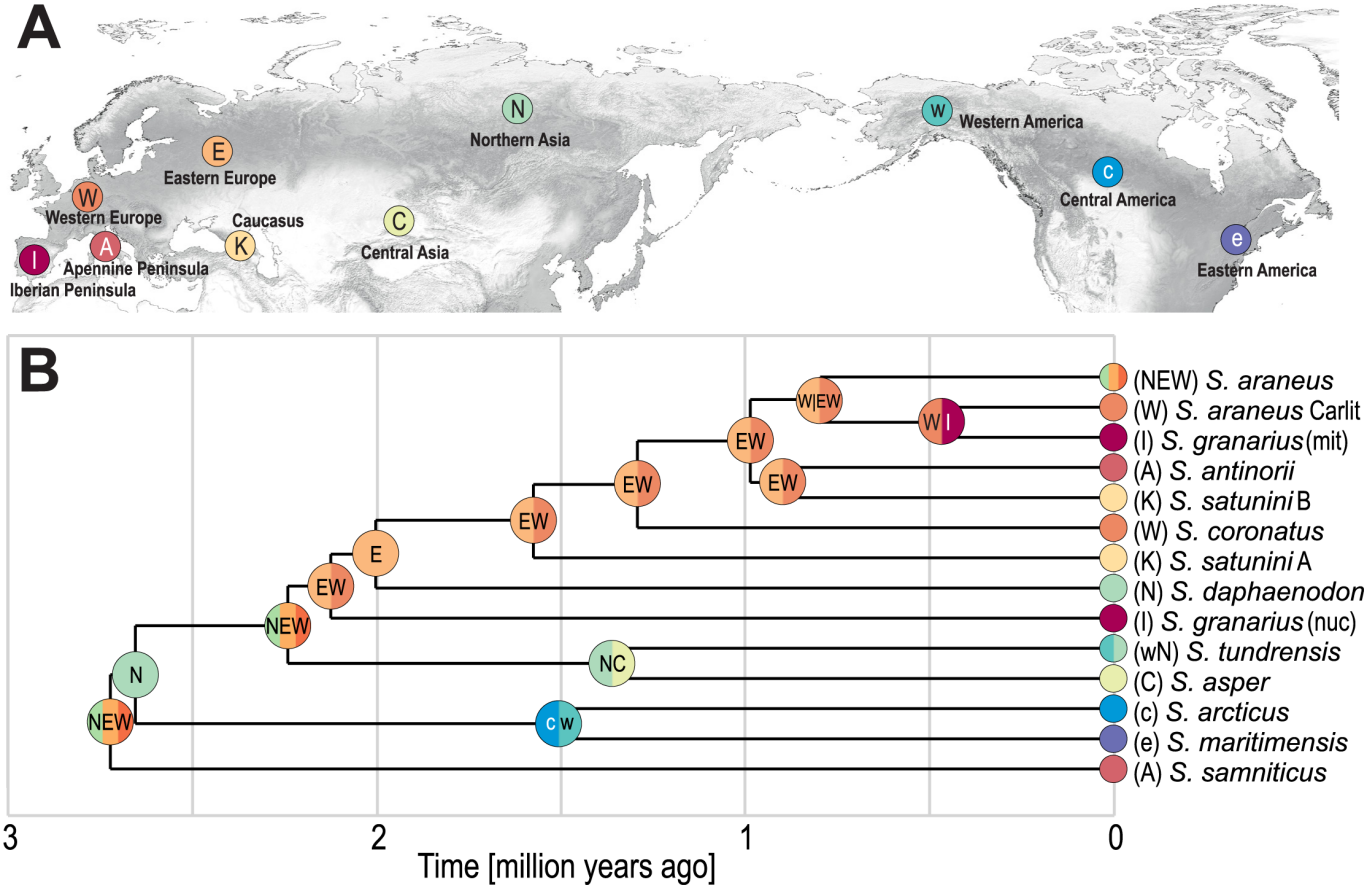
Hypothetical ancestral geographical distribution of *Sorex araneus* lineages. A. Assumed biogeographic regions. B. Timetree assuming two *S*. *granarius* lineages. One diverged before the split of *S*. *daphaenodon*, as is indicated by nuclear data (nuc). The other lineage results from the introgression of mitochondrial DNA from *S*. *araneus* (mit).

The historical biogeography analysis implies that the ancestor(s) of present *S*. *araneus* lineages were widespread in the whole of Eurasia, from Northern Asia to Western Europe, as these regions were selected as the most probable for the basal node (Fig 7). The next node was ascribed to the Northern Asia region, which seems reasonable because the lineage leading to American shrews (*S*. *maritimensis* and *S*. *arcticus*) branches off from this node. A common ancestor of these shrews probably lived in Northern Asia, from where it migrated to Western and Central America across a then-existing land bridge in the place of the current Beringia Strait. The whole Eurasian region was also a diversification centre for further emerged lineages. After the separation of *S*. *tundrensis* and *S*. *asper*, the ancestors of other current *Sorex* lineages occupied the region of Europe.

## Discussion

In order to reconstruct the evolutionary history within the *S*. *araneus* group, we gathered all the available data and studied it using different methods. The phylogenetic tree based on cytochrome b, provided a well-resolved topology recovered by many methods used. Our results are generally in agreement with other phylogenies based on a smaller number of sequences or simpler approaches [12, 34, 36]. However, it was postulated that mitochondrial DNA is subjected to introgression in *Sorex* [12, 34, 36, 37], and as a result it cannot serve as the sole reliable indicator of phylogenetic relationships between shrews. Therefore, we confronted the mtDNA phylogeny with trees based on karyotypic data.

The basal position of *S*. *samniticus* to the members of the *S*. *araneus* group in the mtDNA tree agrees with its placement in the trees based on karyotypic data. Chromosomes of this species are fully acrocentric, have not been subjected to rearrangements and show substantial homologies to the members of *S*. *araneus* group, which implies that *S*. *samniticus* represents the ancestral state in relation to the other taxa [2, 24–26]. This view also agrees with the early divergence of this species in phylogenetic trees based on nuclear markers [34, 37], and differences between it and *S*. *araneus*, *S*. *antinorii* and *S*. *coronatus* in mandibular characters as well as body mass and measures [31].

The subsequently diverging lineages also show the same branching order in the mtDNA and karyotypic trees with the exception of *S*. *granarius*. This species is grouped with significant support with the Carlit race of *S*. *araneus* in the mtDNA tree, whereas in the karyotypic trees, it represents a lineage that evolved at the base of the *S*. *araneus* group. Assuming that the karyotypic trees represent true relationships between *Sorex* lineages, the best explanation for this inconsistency is the introgression of mtDNA from the subpopulation of *S*. *araneus* to *S*. *granarius* [34, 37]. The topology based on the karyotypic data corresponds in principle to the results obtained by Volobouev and Dutrillaux [14] and Taberlet et al. [16] for a smaller number of taxa. The separation of *S*. *granarius* from the other members of the *S*. *araneus* group in the karyotypic trees well agrees with its clear difference in morphology [83]. It is the smallest of the *S*. *araneus* group and also varies from them in many characteristics of skull and mandible. Discriminant and principal component analyses based on these features clearly separated *S*. *granarius* and *S*. *coronatus*, which live in sympatry [84, 85].

Another introgression of mtDNA was suggested to have gone from an ancestor of *S*. *araneus* to the Northern Ciscaucasian population of *S*. *satunini* [12, 36]. In agreement with that, *S*. *satunini* sequences are separated in the mtDNA tree into two clades corresponding to the haplotype A and B (Fig 2). The haplotype A represents most likely an original native lineage because its position in the mtDNA tree is congruent with that in the karyotypic trees (Fig 6). Consequently, the haplotype B would be the introgressed lineage. Most of the applied phylogenetic methods indicated that the donor of the mtDNA could be an ancestor of *S*. *antinorii*, but the tests with alternative placements of *S*. *satunini* did not exclude that this species was introgressed from the *S*. *araneus* lineage or the ancestor of *S*. *araneus* and *S*. *antinorii*, e.g. *S*. *subaraneus* that was widely distributed in Europe in the Pleistocene [86]. In agreement with that, S. *satunini tembotovi* carrying the haplotype B, is identical in morphometric cranial features with *S*. *antinorii* and the model series of *S*. *subaraneus* from German Late Biharian (Early/Middle Pleistocene) [36]. It suggests that not only mtDNA but also other genetic material determining these features could be introgressed. Otherwise, this morphological similarity would be a result of convergence. *S*. *satunini* is a relatively small population characterized by low polymorphism in microsatellite and morphometric mandibular traits, which is a consequence of its small population size, partial isolation, and genetic drift [87]. The same processes could help to maintain the introgressed mtDNA.

The two cases of introgression are confirmed by the species delimitation analyses. *S*. *granarius* and *S*. *araneus* Carlit race were recognized as one cluster by two GMYC methods and the *S*. *satunini* haplotype B and *S*. *antinorii* were grouped together in the multiple GMYC approach. Interestingly, the ABGD method did not separate *S*. *coronatus*, the *S*. *satunini* haplotype B, *S*. *antinorii*, *S*. *granarius* and *S*. *araneus* into individual clusters. This may result from the quite recent differentiation of these taxa and might suggest that some hybridization potential between them was retained. Actually, such a phenomenon was reported for *S*. *antinorii* and *S*. *araneus* [40–44] and the introgression of the Y chromosome from *S*. *coronatus* to *S*. *granarius* was also proposed [34].

**Fig 8:**
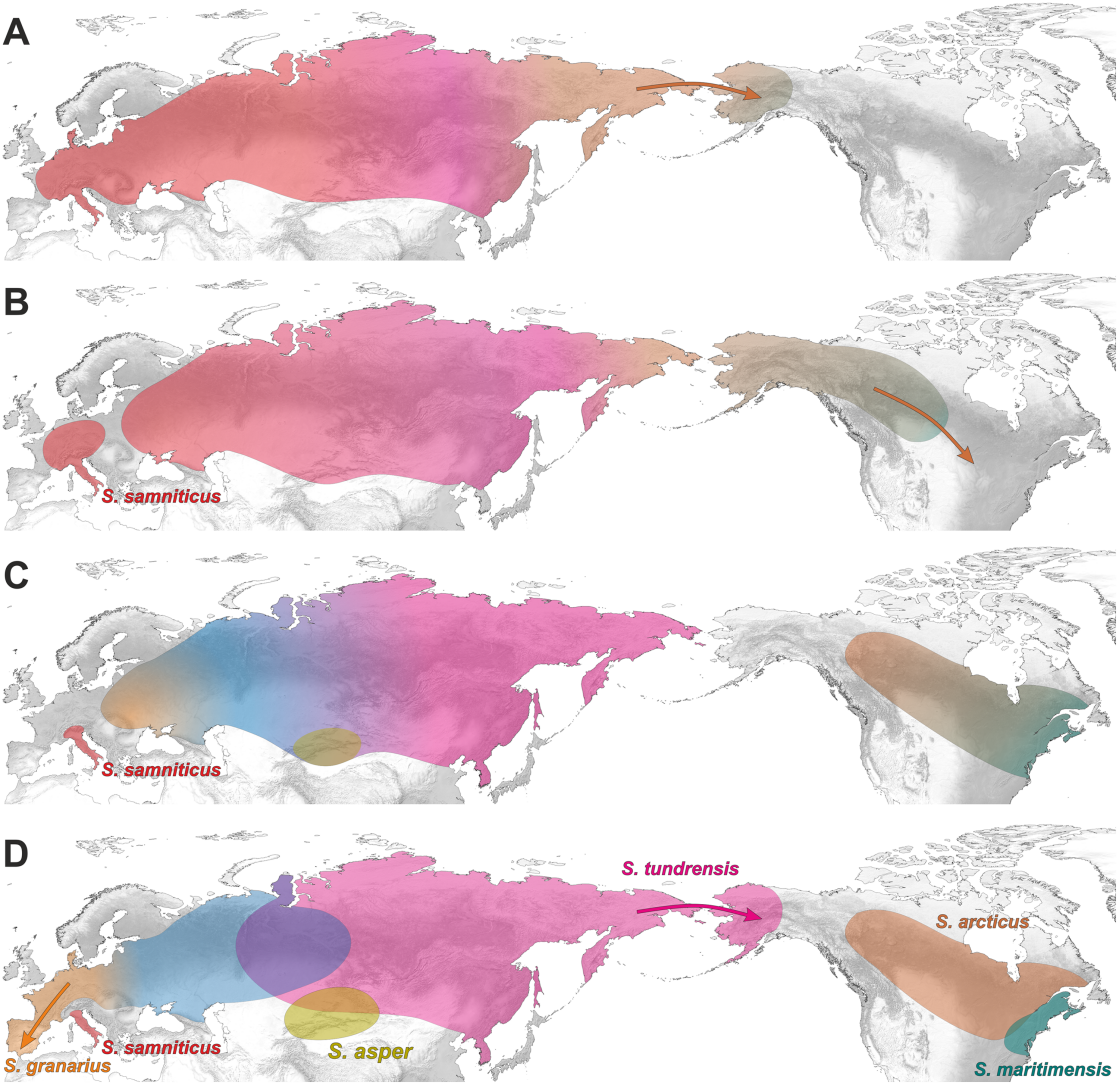
A hypothetical scenario for geographic dispersion of *Sorex araneus* group. A. (3.5–2.7 Mya) An ancestral population is widespread in the whole Eurasia and migrates into Alaska across a land bridge, which existed in the place of the current Beringia Strait. B. (2.7–2.4 Mya) The *S*. *samniticus* lineage emerges within the western European population. This species survived until today in the Apennine Peninsula. The Alaskan population expands into North America. C. (2.4–2.2 Mya) The immigrated population occupies the central and eastern regions of North America. Ancestors of present *Sorex* lineages emerge within the Eurasian and North American populations. D. (2.2–1.4 Mya) The Eurasian population splits into the west and east lineages. The west population spreads into Europe and gives the origin to *S*. *granarius*, restricted currently to Iberian Peninsula. The east population differentiates into *S*. *tundrensis* and *S*. *asper*, whereas the North American population into *S*. *arcticus* and *S*. *maritimensis*. *S*. *tundrensis* migrates into Alaska across Beringia Strait.

**Fig 9:**
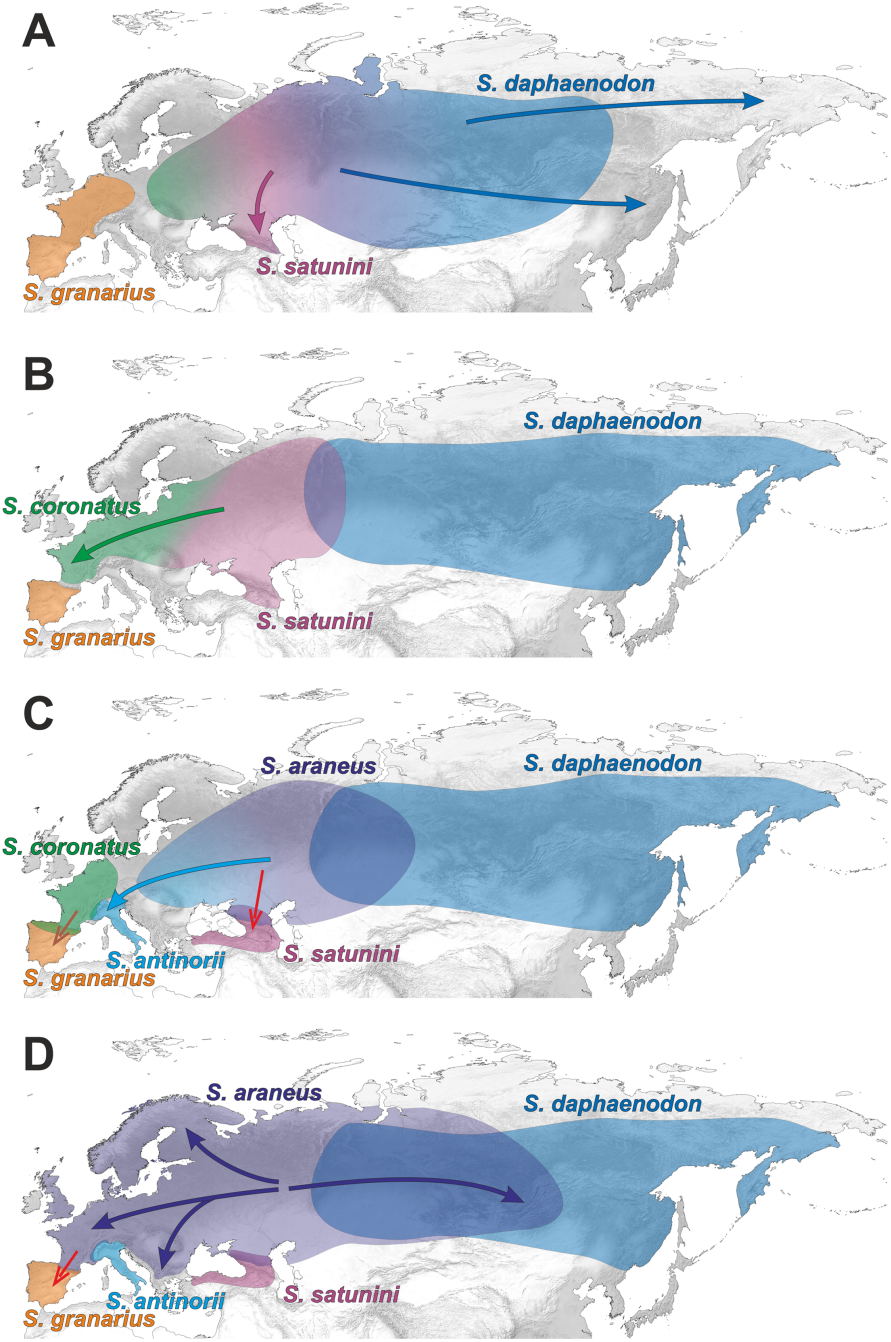
A hypothetical scenario for geographic dispersion of *Sorex araneus* group. **Continuation of** Fig 8. The distribution of *S*. *samniticus*, *S*. *asper* and *S*. *tundrensis* was omitted for clarity. A. (2.0–1.3 Mya) The west Asian population spreads eastward and gives rise to *S*. *daphaenodon*. The *S*. *satunini* lineage emerges in the Caucasus region. B. (1.3–1.0 Mya) *S*. *daphaenodon* spreads to eastern Asia. *S*. *coronatus* originates in Western Europe. C. (1.0–0.8 Mya). The European population differentiates into *S*. *antinorii* and *S*. *araneus*. The southward migration of their ancestor results in the introgression of mitochondrial DNA into the *S*. *satunini* population in the North Caucasus (the red arrow). Similarly, the migration of *S*. *coronatus* into the Iberian Peninsula results in the introgression of the Y chromosome into *S*. *granarius* (the brown arrow). D. (since 0.8 Mya) *S*. *araneus* expands north, west and east. In the Iberian Peninsula, *S*. *araneus* introgresses its mitochondrial DNA into *S*. *granarius* (the red arrow). The distribution of *S*. *coronatus* was omitted for clarity.

Based on available data as well as performed phylogeographic analyses and molecular dating estimates, we decided to propose a hypothetical scenario for the evolution and geographic dispersion of *Sorex araneus* taxa (Figs 8 and 9). Molecular data indicate that Eurasia was the ancestral dominion of the *Sorex* genus [88], which is in agreement with records of their oldest fossils, found in the Late Miocene deposits in Asia [86]. The emergence of the *S*. *araneus* group has also started in the Palearctic, probably in Pliocene about 3 Mya, which agrees well with the geological age of its potential fossil candidates (Fig 5). The oldest representative of this group may be: (1) *S*. *runtonensis* known from the Early (MN17, 2.6–1.9 Mya) to the Late Pleistocene in Europe and Caucasus Mountains [86, 89, 90], or (2) an earlier *Sorex* form Pliocene of Europe (MN15-MN16, 4.2–2.6 Mya), resembling *S*. *runtonensis* [86]. Alternatively, the oldest fossils of this group may represent some early forms described as *S*. *subaraneus* from the Early Pleistocene deposits (MN17, 2.6–1.9 Mya) of France or even Pliocene (MN15, 4.2–3.2 Mya) of Slovakia [86]. Under this name, there are also described morphologically diverse forms found in sediments from the whole Pleistocene in different regions of Europe. Both *S*. *runtonensis* and *S*. *subaraneus* are highly polymorphic and could be regarded as a morphometrical continuum of the same species. They may represent ancestors or old remains of the lineages leading to current species. Other detailed studies are required to solve the taxonomic position of these two taxa [89].

The ancestral populations of the *S*. *araneus* group were probably widespread in the whole of Eurasia. A relict lineage that diverged as the first about 2.7 Mya and survived in the Apennine Peninsula up to the present is represented by *S*. *samniticus* (Fig 8A and 8B). Unfortunately, fossil remains of this taxon are poorly represented [86]. They were reported by Kostakis et al. [91] only for the Late Pleistocene (MIS2) deposits from the Baccano maar area in central Italy. However, it cannot be excluded that shrew forms described under the name of *S*. *runtonensis* and *S*. *subaraneus* known since the Early (ca. 1.1 Mya) and Late Biharian (ca. 0.75 Mya) of Italy, [92], represent the *S*. *samniticus* lineage implying its continuity in the Apennine Peninsula.

The extant *S*. *arcticus* and *S*. *maritimensis*, lineages emerged also within the Early Pleistocene *S*. *runtonensis* or *S*. *subaraneus* forms, likely in North-eastern Asia and then migrated across the Beringia Strait into North America about 2.7 Mya (Fig 8). This ancestral population split about 1.5 Mya into the current species, which occupy central and eastern parts of the continent, respectively. This dating agrees with the oldest fossils of *S*. *arcticus* dated to the Late Irvingtonian (Early/Middle Pleistocene, 0.900–0.690 Mya) of Colorado and Virginia in USA [93, 94]—Fig 5. In 3 Mya, the global sea level started to fall below the current one due to Pleistocene glaciations. In the considered period, the level decreased even by 75 m [95], which caused the emergence of the Beringia Strait and made the fauna migrations possible.

About 2.2 Mya, Eurasian *Sorex* differentiated into the west and east lineages (Fig 8D). The extant descendant of the former may be *S*. *granarius*, restricted currently to the Iberian Peninsula, whereas the latter evolved into *S*. *tundrensis* and *S*. *asper*. *S*. *tundrensis* has been more evolutionary successful because it inhabited the whole Eastern Palearctic and migrated into Alaska across Beringia Strait. Its ancestor could be *S*. *runtonensis* (from the Early to the Late Pleistocene), with which it shares similar mandibular size and proportions [96]. *S*. *tundrensis* is an eurytopic taxon and has diversified into several lineages since ca. 0.870 Mya, which corresponds well with its fossil records known from Moneron Island and dated to the Early Pleistocene (ca. 0.700 Mya) [14, 97]. The diversification was associated with Pleistocene glacial cycles producing space-time conditions that enabled the isolation of populations [13, 39]. In agreement with the strong geographic population structure, the species delimitation methods recognized within *S*. *tundrensis* several clusters. Although some cytogenetic studies proposed the existence of several species within the Holarctic shrew [17], additional genetic and morphological studies of its populations are required to determine their subspecies or even species status [19, 39]. Ecological niche modelling and ancient DNA analysis indicate that *S*. *tundrensis* was widely distributed also across Europe during the Last Glacial Maximum [98].

About 2 Mya additional Asian and European lineages emerged (Fig 9A and 9B). The former evolved into *S*. *daphaenodon* that spread across Central and Eastern Asia. It must have reached the Far East before 900 kya because its oldest remains are dated to this time in Kolyma Lowland deposits [63, 64]. In turn, the European populations differentiated about 1.6–1.3 Mya into the lineages of Caucasian *S*. *satunini* and Western European *S*. *coronatus* (Fig 9B). Remains of *S*. *satunini* were described from the Middle Pleistocene deposits of Northern Caucasus dated to about 0.400 Mya [65, 66]. Fossils resembling *S*. *coronatus* are known from the Middle Pleistocene of France [86]. Some of them are dated to MIS 15 (0.621–0.563 Mya) [99]. *S*. *coronatus* invaded the Iberian Peninsula and hybridized with *S*. *granarius*, which resulted in the introgression of Y chromosome to the Iberian shrew [34]—Fig 9C.

The European populations separated then into the ancestors of *S*. *antinorii* and *S*. *araneus* about 990 kya (Fig 9C). Some Middle and Late Pleistocene shrews assigned to *S*. *subaraneus* may represent their ancestors [86]. *S*. *antinorii* confined its distribution into the Apennine Peninsula, which was associated with the glaciation of Alps in the Late Pleistocene and the isolation of its populations into refugial areas [100, 101]. The fossil records of *S*. *antinorii* are poorly represented. Its remains were reported for the Late Pleistocene (MIS2) deposits from the Baccano maar area in central Italy [91]. However, all fossil remains from the Apennine Peninsula ascribed to *S*. *araneus* should be most probably assigned to *S*. *antinorii* [91]. Consequently, the oldest fossils of these forms known from the late Early Toringian of Italy (ca. 0.35 Mya) [92] can in fact represent the *S*. *antinorii* lineage. It agrees with our estimation of *S*. *antinorii* differentiation dated to about 0.56 Mya.

The ancestral population of the current *S*. *araneus* could occupy Eastern Europe as it is suggested by the basal placement of Russian sequences in the phylogenetic trees (S1 and S2 Figs). Then it started its expansion into Northern, Western and Southern Europe as well as into Western and Central Asia (Fig 9C). These results imply that the oldest fossils of *S*. *araneus* should be found in Eastern Europe. However, fossil shrews from this region are poorly represented and fragmentary remains can be misclassified, e.g. *S*. *araneus* may be confused with *S*. *subaraneus* and *S*. *coronatus* [86]. So far the oldest (the Early Pleistocene) remains of *S*. *araneus* were reported in regions located in Eastern Europe, i.e. Romania and Ukraine but also in Czech Republic and Netherlands [102, 103].

The ancestor of *S*. *antinorii* and *S*. *araneus* migrated into North Caucasus and introgressed mitochondrial DNA to *S*. *satunini* population [12, 36]. The other introgression happened again in the Iberian Peninsula, where *S*. *araneus* introgressed its mitochondrial DNA into *S*. *granarius* [34, 37]–Fig 9D. The introgressed mtDNA lineages separated from their maternal ancestors about 900 kya and 465 kya, respectively.

Our results imply that the diversification rate of new lineages within the *S*. *araneus* group is significantly correlated with the variance in climatic fluctuations described by the δ^18^O curve (Fig 5). The rate rapidly increased in the Middle and Late Pleistocene, since 1 Mya, when climate oscillations became most pronounced. The diversification of shrews can be explained by the influence of climatic and environmental conditions on populations according to a contraction-expansion model [96, 104, 105]. During glacial periods, larger populations were fragmented into smaller isolated ones, which were subjected more keenly to bottlenecking and genetic drift. Consequently, this caused the evolution of distinct lineages with different genetic pools. The new populations could next expand during warmer periods (interglacials). Repeated extensions and regressions could increase the genetic differentiation and speciation.

The presented results are based on cytochrome b, quite a reliable and repeatedly tested mitochondrial marker in shrews. However, additional sequences from the mitochondrial genome could be used to confirm or reject the observed relationships between the studied taxa, especially the proposed introgression events. Similarly, the relationships and evolutionary scenario deduced from the karyotypic data could be verified based on other nuclear markers. The already-used fragments of nuclear genes *BRCA1* and *ApoB* as well as Y chromosome loci did not provide fully resolved phylogenetic trees [37]. Therefore, there is a need to search for additional nuclear markers to confirm relationships based on chromosomal whole-arm rearrangements.

To fully reconstruct the history of *S*. *araneus* group, also a revision of all fossils ascribed to *S*. *subaraneus*, *S*. *araneus* and related shrews is necessary [86], including ancient DNA analysis, because fossil remains assigned to current species can represent other forms, and fossils described under different names can belong to the present lineages, e.g. *S*. *macrognathus* appears to be a large ectomorph of *S*. *araneus* rather than a distinct species [98].

## Supporting information

S1 Table Nucleotide substitution models applied in phylogenetic inferring for three alignment sets. (PDF)

S2 Table Parameters and the Akaike information criterion (AIC) for different diversification models fitted to the time-calibrated phylogeny. (PDF)

S3 Table The oldest fossil records of selected members of *Sorex araneus* group. (PDF)

S1 Fig MrBayes tree based on 1011 bp cytochrome b alignment for *Sorex araneus*. (PDF)

S2 Fig MrBayes tree based on 1140 bp cytochrome b alignment for *Sorex araneus*. (PDF)
