# Supplementary material for: Evolutionary history and phylogeographic relationships of shrews from Sorex araneus group
Source: PLoS One. 2017 Jun 26;12(6):e0179760. doi: 10.1371/journal.pone.0179760 (PMC5484494; doi:10.1371/journal.pone.0179760)
Supplement: S1 Table — (PDF) [file pone.0179760.s001.pdf]

S1 Table. Nucleotide substitution models applied in phylogenetic inferring for three alignment sets.

Program	Partition	Sequence alignment sets		
		(1) 111 taxa, 1011 bp	(2) 140 taxa, 1011 bp	(3) 93 taxa, 1140 bp
	1. codon position	mixed+Γ+I	mixed+Γ+I	mixed+Γ+I
MrBayes	2. codon position	mixed+Γ+I	mixed+Γ+I	mixed+Γ+I
	3. codon position	mixed+Γ+I	mixed+Γ+I	mixed+Γ+I
	1. codon position	TN+Γ	HKY+Γ	HKY+Γ
TreeFinder	2. codon position	HKY{Ts=3, Tv=1}+Γ	HKY{Ts=3, Tv=1}+Γ	HKY{Ts=3, Tv=1}
	3. codon position	GTR+Γ	TN+Γ	J2+Γ
	1. codon position	011020+Γ	NA	NA
Beast	2. codon position	F81	NA	NA
	3. codon position	012343+Γ	NA	NA
morePhyML & PAUP	All codon positions	012030+Γ+I	HKY+Γ	HKY+Γ
