# Supplementary material for: Evolutionary history and phylogeographic relationships of shrews from Sorex araneus group
Source: PLoS One. 2017 Jun 26;12(6):e0179760. doi: 10.1371/journal.pone.0179760 (PMC5484494; doi:10.1371/journal.pone.0179760)
Supplement: S2 Table — (PDF) [file pone.0179760.s002.pdf]

S2 Table. Parameters and the Akaike information criterion (AIC) for different diversification models fitted to the time-calibrated phylogeny.

Model description	Model	Parameters	AIC	ΔAIC
Constant-rate models	Pure birth (Yule)	λ=1.760	-723.89	35.48
	Constant-rate birth–death (BD)	λ=2.598<br>μ=1.643	-730.44	28.93
Variable-rate models assuming different speciation rates within specified time intervals	Yule2rate	λ1=1.891<br>t1=0.048<br>λ2=0.375	-745.77	13.6
	Yule3rate	λ1=0.662<br>t1=0.796<br>λ2=2.993<br>t2=0.130<br>λ3=1.021	-759.25	0.12
	Yule4rate	λ1=0.662<br>t1=0.796<br>λ2=2.993<br>t2=0.130<br>λ3=1.433<br>t3=0.048<br>λ4=0.375	-759.37	0.00
	Yule5rate	λ1=0.662<br>t1=0.796<br>λ2=2.697<br>t2=0.402<br>λ3=337.53<br>t3=0.402<br>λ4=3.100<br>t4=0.130<br>λ5=1.021	-759.08	0.30
Variable-rate model assuming decreasing diversification through time in relation to increasing diversity with exponential fitting	DDX	λ=0.583<br>x=-0.308	-733.28	26.10
Variable-rate model assuming decreasing diversification through time in relation to increasing diversity with logistic fitting	DDL	λ=1.760<br>k=1840527	-721.89	37.48
Variable-rate model assuming decreasing diversification through time caused by decreasing speciation rate	SPVAR	λ=2.608<br>μ=1.647<br>k=0.001	-728.41	30.97
Variable-rate model assuming decreasing diversification through time caused by increasing extinction rate	EXVAR	λ=2.597<br>μ=1.641<br>z=889.05	-728.44	30.93
Variable-rate model assuming decreasing diversification through time caused by decreasing speciation rate and increasing extinction rate	BOTHVAR	λ=2.609<br>μ=1.648<br>k=0.001<br>z=886.94	-726.41	32.97

λ, speciation; μ, extinction; t, time of rate-shift in millions of years ago; x, parameter controlling the magnitude of the rate change; K, parameter analogous to the ‘carrying capacity’ parameter in population ecology; k, parameter of the exponential change in speciation rate; z, parameter of the exponential change in extinction rate.
