# Supplementary material for: Evolutionary history and phylogeographic relationships of shrews from Sorex araneus group
Source: PLoS One. 2017 Jun 26;12(6):e0179760. doi: 10.1371/journal.pone.0179760 (PMC5484494; doi:10.1371/journal.pone.0179760)
Supplement: S3 Table — (PDF) [file pone.0179760.s003.pdf]

S3 Table. The oldest fossil records of selected members of *Sorex araneus* group.

Species name	Age	Locality	Reference
Sorex antinorii	Late Early Toringian (0.350 Mya)	Loara and Sant'Agostino (Italy)	[1,2]
Sorex arcticus	Late Irvingtonian (0.900-0.690 Mya)	Hansen Bluff (Alamosa Co., Colorado, USA), Trout Cave (Pendleton Co., Virginia, USA)	[3,4]
Sorex coronatus	Middle Pleistocene (MIS 15, 0.621-0.563 Mya)	Carpentier Quarry (Abbeville, France)	[5,6]
Sorex daphaenodon	Olyorian formation (0.800-0.950 Mya)	Kolyma Lowland (Yakutia, Russia)	[7,8]
Sorex samniticus	Early Biharian (1.100 Mya)	Monte Peglia (Italy)	[2]
Sorex satunini	Middle Pleistocene (0.360 Mya)	Treugolnaya Cave (Northern Caucasus, Russia)	[9,10]
Sorex tundrensis	Early Pleistocene (0.700 Mya)	Moneron Island (Russia)	[11,12]

1. Kostakis T, Marcolini F, De Rita D, Conti M, Esu D (2011) Three Late Pleistocene small mammal faunas from the Baccano maar (Rome, central Italy). Bollettino della Società Paleontologica Italiana Modena 50 103-110.
2. Kotsakis T, Abbazzi L, Angelone C, Argenti P, Barisone G, et al. (2003) Plio-Pleistocene biogeography of Italian mainland micromammals. Deinsea 10: 313-342.
3. Rogers KL, Repenning CA, Forester RM, Larson EE, Hall SA, et al. (1985) Middle Pleistocene (Late Irvingtonian: Nebraskan) climatic changes in south-central Colorado. National Geographic Research 1: 535-563.
4. Kirkland G, Schmidt D (1996) Sorex arcticus. Mammalian Species 524: 1-5.
5. Rzebik-Kowalska B (2009) Biodiversity of Polish fossil insectivores (Erinaceomorpha, Soricomorpha, Insectivora, Mammalia) compared to the European and global faunas. Institute of Systematic and Evolution of Animals, Polish Academy of Sciences, Kraków.
6. Antoine P, Moncel M-H, Limondin-Lozouet N, Locht J-L, Bahain J-J, et al. (2016) Palaeoenvironment and dating of the Early Acheulean localities from the Somme River basin (Northern France): New discoveries from the High Terrace at Abbeville-Carrière Carpentier. Quaternary Science Reviews 149: 338-371.
7. Sher A (1974) Pleistocene mammals and stratigraphy of the Far Northeast USSR and North America. International Geology Review 16: 1–284.
8. Virina E, Zazhigin V, Sher A (1984) Paleomagnetic characteristic of the type sites of the Olyorian Faunal complex (Kolyma Lowland). Izvestia of Russian Academy of Science, Sergeol 11: 61–71.
9. Zaitsev MV, Baryshnikov GF (2002) Pleistocene Soricidae (Lipotyphla, Insectivora, Mammalia) from Treugolnaya Cave, Northern Caucasus, Russia. Acta Zoologica Cracoviensia 45: 283-305.
10. Zaitsev MV, Osipova V (2005) Taxonomy of Middle and Late Pleistocen shrews from the Northern Caucasus. In: Merritt JF, Churchfield S, Hutterer R, Sheftel BI, editors. Advances in the Biology of Shrews II New York: International Society of Shrew Biologists. pp. 49–62.
11. Volobouev V, Dutrillaux B (1991) Chromosomal evolution and phylogenetic relationships of the Sorex araneus-arcticus species group. In: Hausser J, editor. The cytogenetics of the Sorex araneus group and related topics Proceedings of the ISACC's Second International Meeting Mémoires de la Société Vaudoise des Sciences Naturelles. Zürich, Schweiz: Ein Dienst der ETH-Bibliothek. pp. 131-139.
12. Okhotina MV (1983) A taxonomic revision of Sorex arcticus Kerr, 1792 (Soricidae, Insectivora). Zoologischeskii Zhurnal 62: 409-417.
