# Supplementary material for: Evolutionary history and phylogeographic relationships of shrews from Sorex araneus group
Source: PLoS One. 2017 Jun 26;12(6):e0179760. doi: 10.1371/journal.pone.0179760 (PMC5484494; doi:10.1371/journal.pone.0179760)

S1 Fig. MrBayes tree for the alignment of cytochrome b sequences with the length of 1011 bp from *Sorex araneus* and *S. coronatus* used as an outgroup. Numbers at nodes, in the order shown, correspond to: posterior probabilities estimated in MrBayes (MB), support values obtained by the Local Rearrangements-Expected Likelihood Weights (LR-EWL) method in TreeFinder and the approximate likelihood ratio test based on a Shimodaira-Hasegawa-like procedure (SH) in morePhyML, as well as bootstrap values obtained in TreeFinder (TF), PhyML (Ph) and PAUP by neighbour joining (NJ) method. Values of the posterior probabilities and bootstrap percentages lower than 0.50 and 50%, respectively, were omitted or indicated by a dash "-". The length of branches leading to *S. coronatus* was reduced by half. The colours correspond to geographical locality of samples. In square brackets, names of races and karyotypic groups were included if they were known: EEKG (East European karyotypic group), NEKG (North European karyotypic group) and WEKG (West European karyotypic group).

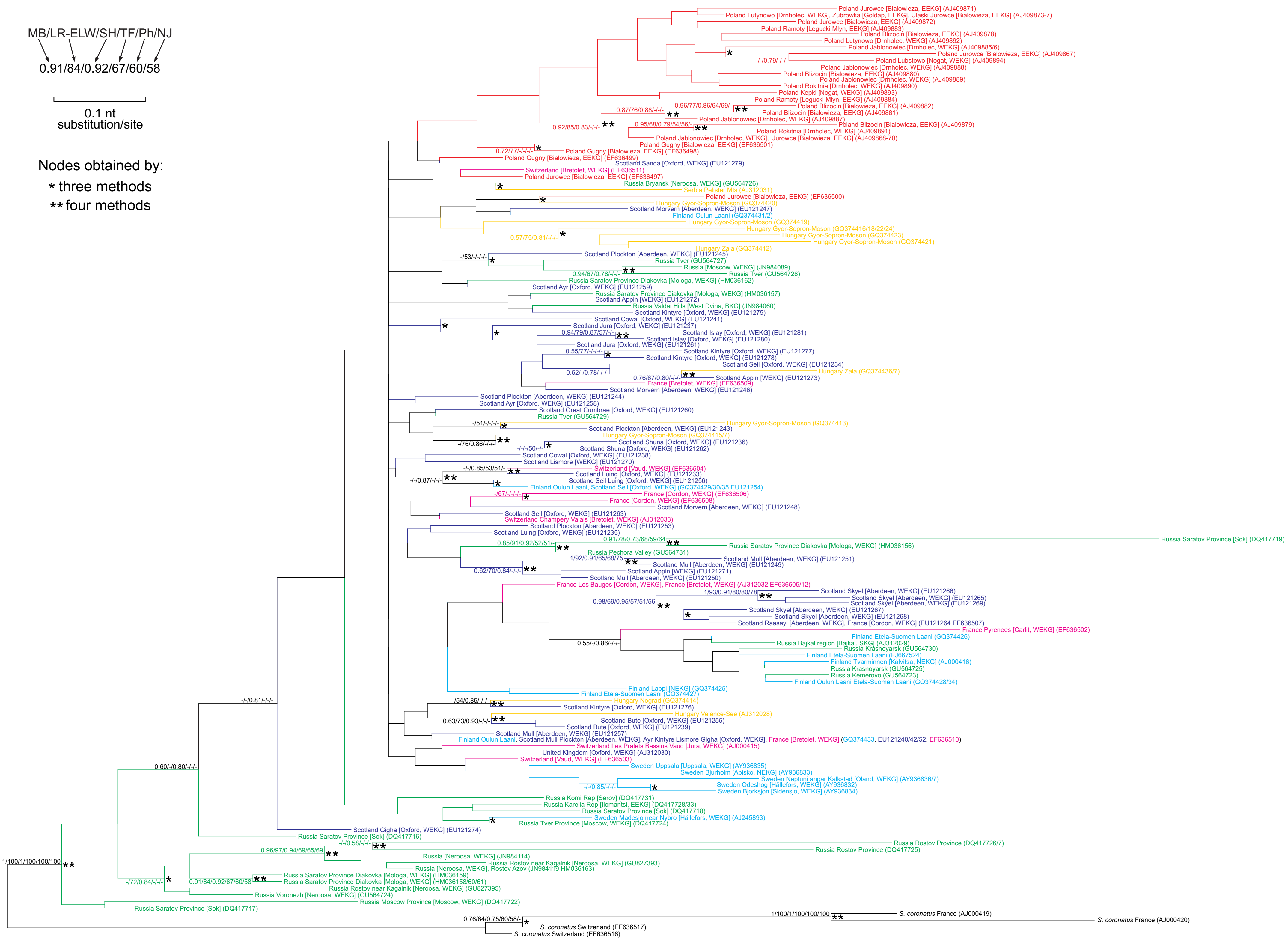

Supplement: S1 Fig — (PDF) [file pone.0179760.s004.pdf]
